# Developing a Thai User Interface Terminology for Systematized Nomenclature of Medicine Clinical Terms Implementation in Primary Care: Cross-Sectional Content Coverage Analysis

**DOI:** 10.2196/80039

**Published:** 2026-03-09

**Authors:** Nat Tangchitnob, Wanchana Ponthongmak, Boonchai Kijsanayotin, Oraluck Pattanaprateep, Sithakom Phusanti, Pongsakorn Atiksawedparit, Kamonporn Suwanthaweemeesuk, Jirayus Siangfu, Gareth J McKay, John Attia, Ammarin Thakkinstian

**Affiliations:** 1 Department of Clinical Epidemiology and Biostatistics, Faculty of Medicine, Ramathibodi Hospital, Mahidol University, Rama 6 Road, Ratchathewi, Bangkok, 10400, Thailand, 0833744997; 2 Thai Health Information Standards Development Center, Health Systems Research Institute, Ministry of Public Health, Bangkok, Thailand; 3 Chakri Naruebodindra Medical Institute, Faculty of Medicine Ramathibodi Hospital, Mahidol University, Bangkok, Thailand; 4 Chulabhorn Hospital, Chulabhorn Royal Academy, Bangkok, Thailand; 5 Centre for Public Health, Queen's University Belfast, Belfast, United Kingdom; 6 Centre for Clinical Epidemiology and Biostatistics, School of Medicine and Public Health, University of Newcastle Australia, Newcastle, Australia

**Keywords:** standards and interoperability, Systematized Nomenclature of Medicine Clinical Terms, SNOMED CT, eHealth infrastructure, clinical terminology, natural language processing, concept coverage

## Abstract

**Background:**

Primary care in Thailand often uses mixed Thai-English free-text documentation for diagnoses and clinical problems, limiting standardization, interoperability, and secondary data use. Clinical terminologies like Systematized Nomenclature of Medicine Clinical Terms (SNOMED CT), a comprehensive reference terminology, can bridge this gap through the use of structured clinical data. Developing and mapping a local user interface terminology (UIT) is one of the key strategies for implementing SNOMED CT in real-world clinical settings.

**Objective:**

This study aimed to develop a Thai UIT derived from frequently used terms in real-world primary care practice, map these terms to SNOMED CT concepts, and evaluate the extent of concept coverage.

**Methods:**

Frequently used clinical terms were extracted from outpatient medical records from the family, emergency, and internal medicine departments using a customized tokenization method, N-gram analysis, and expert review. This process yielded 2054 Thai-specific terms. All terms were normalized and mapped to SNOMED CT through manual expert-driven and semiautomated tools. Unmapped terms were subsequently analyzed to identify mapping barriers and solutions.

**Results:**

Of the 2054 Thai-specific terms, 2012 were successfully mapped to 2041 (97.98%) SNOMED CT concepts, including 1781 (85.50%) fully, 123 (5.90%) broader, 56 (2.69%) narrower, 81 (3.89%) inexact mappings, and 42 (2.02%) remained unmapped. Most mappings were one-to-one (1984), with 28 terms mapped to multiple concepts (57), covering 1486 unique SNOMED CT concepts. The remaining 42 unmapped terms were mostly due to culturally specific expressions or concepts not yet represented in SNOMED CT. These were categorized for potential postcoordination, exclusion, or national extension development.

**Conclusions:**

This study demonstrates the feasibility of developing a Thai UIT mapped to SNOMED CT and describes mapping challenges. The resulting UIT enhances semantic clarity in clinical documentation and supports better interoperability, clinical decision-making, and health data analytics within Thailand’s health care system.

## Introduction

Health care provision is a knowledge-intensive industry, but the quality and accessibility of health care data often fail to adequately meet these needs [1]. This is partly due to the data collection process, which commonly takes place in a fast-paced, high-pressure point-of-care environment [2], leading to the generation of data sufficient to meet the immediate clinical tasks but often lacking the completeness, standardization, and structure necessary for secondary use, for example, research, that drives health care improvement [3,4]. This limitation may compromise patient safety, care quality, and the ability to generate actionable insights [2,5]. To harness the full potential of health care data, it is essential to prioritize structured data collection that supports timely and effective use across the health care ecosystem [3,4]. Standardized clinical terminology, structured representations of clinical concepts understandable to both humans and computer systems, is an essential technology improvement required for developing reusable data that enables interoperable health care delivery [6].

Because clinical terminology must be comprehensive and support multilingual use, the Systematized Nomenclature of Medicine Clinical Terms (SNOMED CT) has been widely adopted internationally as a reference terminology standard that is indispensable for standardized information exchange across clinical care and research settings [7]. SNOMED CT adopts a concept-oriented structure; each unique clinical idea is represented by a concept identified by a language-independent, machine-readable unique identifier.

SNOMED CT consists of 3 core components, including concepts, descriptions, and relationships. Concepts represent the underlying clinical meaning; descriptions provide human-readable terms in multiple languages; and relationships define how concepts are linked within the ontology. A single concept can have multiple descriptions, enabling flexible, precise, and multilingual representation of clinical ideas. Relationships form a poly-hierarchical graph structure to express, distinguish, and connect each clinical concept, maximizing practicality while minimizing ambiguity [8]. For example, the concept “Nodule of lung (disorder)” can be described by multiple terms across languages (eg, English, French, and Swedish), and its meaning is defined through relationships as the child of both “Lesion of lung (disorder)” and “Lung mass (finding),” the associated morphology “Nodule (morphologic abnormality),” and the finding site “Lung structure (body structure).” This structure enables consistent clinical data representation and supports semantic interoperability. Successfully implementing SNOMED CT not only improves data quality and standardization of clinical data but also enhances the quality of health care through more efficient data use for both clinical and research applications [9,10]. While SNOMED CT offers a robust, language-independent conceptual framework, it does not automatically capture the variability of clinical expressions used in routine practice, particularly in multilingual or mixed-language documentation environments.

User interface terminology (UIT) refers to the set of terms clinicians use during routine clinical documentation. It consists of user-friendly and context-specific expressions that reflect the natural phrasing and cognitive patterns of clinical practice [11]. UITs are typically designed to be used by a defined user group and certain settings, prioritizing efficiency and usability [12]. Within the Open Medical Record System (OpenMRS; OpenMRS Inc) ecosystem, many implementations rely on Columbia International eHealth Laboratory (CIEL), an openly maintained UIT that serves as a common starting point for clinical data capture in OpenMRS deployments [13]. CIEL supports user-friendly clinical data capture while providing mappings to international reference terminologies (including SNOMED CT) [14], enabling the harmonization of locally meaningful data for use with global standards, facilitating secondary data use, interoperability, and research usability [3,4]. Combining the advantages of both terminologies leverages their complementary strengths, enhancing communication accuracy, data quality, and adoption among clinicians, while providing semantic precision, computability, and cross-system interoperability [10,11]. Together, they facilitate the standardization of a clinical narrative into more structured data [15,16].

Despite its advantages, implementing SNOMED CT presents challenges, particularly in non–English-speaking contexts [7,10,17]. Studies investigating the performance of SNOMED CT in multilingual contexts report 2 important indicators. Concept coverage refers to the proportion of source clinical ideas that can be represented by a concept, while term coverage refers to the proportion of source terms included in the SNOMED CT description, reflecting comprehensiveness of concept and the usability of descriptions, respectively [18]. For example, European-based studies demonstrate varying degrees of success in translation and implementation efforts. A fully translated Swedish version of SNOMED CT achieved good concept coverage (87%) but lower term coverage (47%), reflecting limited alignment with real-world clinical language [19]. In contrast, a German UIT without the use of SNOMED CT reached comparable term coverage (46%) despite the lack of codes in some key semantic areas, such as procedures, devices, or genes [16,19]. These findings suggest that constructing or adopting language-specific UITs, linked to SNOMED CT’s richer semantics and logical structure, is urgently needed and more effective than simply relying on direct translation [10,15].

Thailand became a member of SNOMED International in 2022, signaling a national commitment to adopt SNOMED CT as the reference terminology for addressing the country’s interoperability and health care data quality challenges [20]. Since joining, SNOMED CT codes have been incorporated into the national reporting and reimbursement datasets for diagnostic data. In clinical practice, a few health care institutes have begun integrating SNOMED CT’s abundant descriptions into their diagnosis entry systems. This approach complements the previous process, in which physicians manually selected *International Classification of Diseases (ICD)* codes. However, the potential benefits of adopting SNOMED CT (eg, improved data granularity, semantic interoperability, and enhanced secondary data use) have yet to be fully realized across the country [9,10]. A prior study in Thailand attempted to auto-map Thai discharge summary terms to *ICD* codes through SNOMED CT descriptions but excluded all non-English characters, limiting its ability to interpret locally used abbreviations and mixed-language expressions common in Thai clinical documentation [21].

This study was conducted with the following 3 main objectives. First, to develop a Thai-language UIT derived from real-world clinical practice. Using a dynamic corpus of clinical texts, we apply a term collection methodology that integrates clinical expertise with natural language processing (NLP) techniques. The resulting UIT is expected to facilitate and support standardized data entry, enhance data quality, and improve user acceptance of SNOMED CT within Thailand’s health care system.

Second, to evaluate the content coverage of SNOMED CT, defined as the proportion of clinical ideas in the Thai context that can be represented through international reference terminology.

Third, to perform mapping analysis of partially mapped and unmapped terms in order to identify potential areas where SNOMED CT or the Thai national extension may need further refinement to better represent Thai health care data.

## Methods

### Data Source

Data used for the development of the UIT were retrospectively retrieved from the chief complaint and present illness sections of electronic medical records (EMRs) at Chakri Naruebodindra Medical Institute, Ramathibodi Hospital, Mahidol University, covering the period from January 2019 to April 2023. Data were obtained from outpatient clinics in the family medicine (FM) and emergency departments, representing primary care settings. To enhance lexical diversity and coverage, additional data were obtained from the high-volume internal medicine (IM) department. Records lacking both the chief complaint and present illness sections were excluded from the study.

### UIT

In this study, UIT was defined as a set of clinically relevant terms used by clinicians to express their clinical ideas within the EMRs. These terms can be presented in either the semistructured or unstructured free-text entries. To qualify as UIT, the terms must contain adequate meaning, clarity, and clinical specificity to support accurate documentation and enable secondary uses, as assessed by medical doctors (eg, “เจ็บหน้าอก” [chest pain], “อาเจียนเป็นเลือด” [vomiting blood], “โรคหัวใจขาดเลือด” [ischemic heart disease]), whereas terms that were vague or noninformative (eg, “1 เดือน pta” [1 month prior to admission] or “without evidence of”) were not considered UIT.

**Figure 1.**
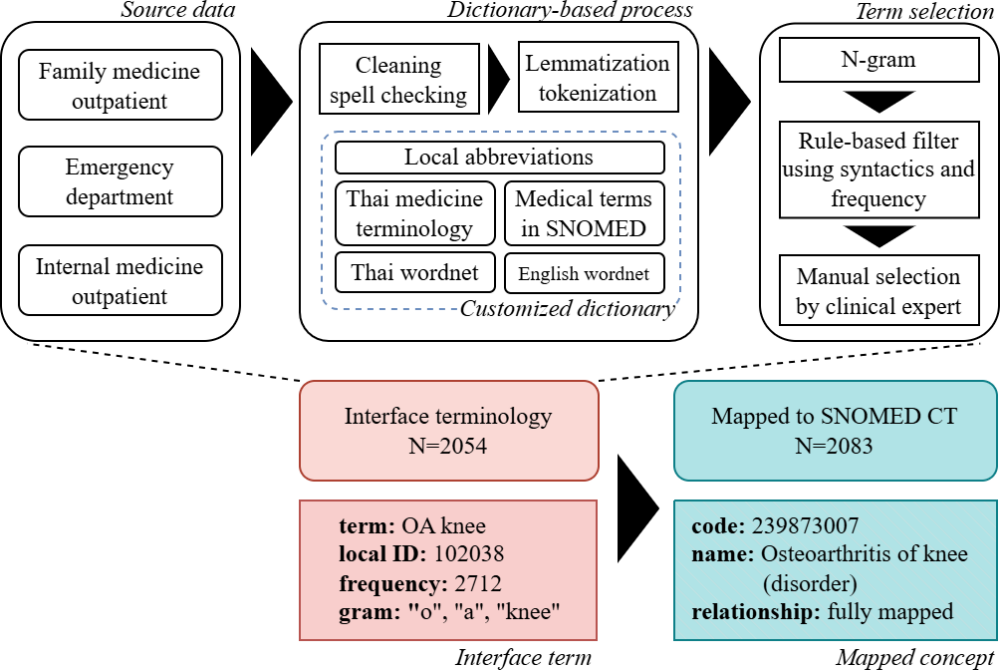
Summary overview of the mapping process. Collecting data from all sources (left upper box). Turning free text to N-gram using a customized dictionary from 5 sources (middle upper box). Interface terms selected by term frequency and experts (right upper box). Indexing the interface terms (pink). Mapping to the semantically equivalent SNOMED CT concept (blue). OA: osteoarthritis; SNOMED CT: Systematized Nomenclature of Medicine Clinical Terms.

The content of the UIT was harvested from the free-text documents produced during actual patient encounters. Clinician-entered free-text data (chief complaint and present illness) from the EMRs were preprocessed and transformed into clinical terms using NLP techniques, including cleaning, tokenization using our customized method, and N-gram generation. Term frequencies were then calculated, and terms meeting predefined threshold criteria were included in the UIT. The overall process is illustrated as follows (see Figure 1):

### Text Preprocessing and Tokenization

Free-text data were preprocessed using the Python library for Thai NLP (ie, PyThaiNLP version 4.0.2) [22], which normalized texts by applying case normalization, correcting misplaced Thai tone marks and vowels, removing punctuation, and eliminating duplicate entries (eg, repeated sentences from prior visits). This process removed approximately 11.4% of the total characters, improving data consistency and validity. The code used for implementing text preprocessing and tokenization is provided in the GitHub repository [23].

Because Thai is a continuous-script language, which has no word and sentence boundaries (eg, “โรคหัวใจขาดเลือด” [ischemic heart disease] consists of 4 lexical units: “โรค”(disease), “หัวใจ”(heart), “ขาด”(deficiency), “เลือด”(blood)), standard Thai tokenizers trained on general Thai corpora perform poorly, particularly for medical text [24]. We therefore used a customized maximum matching algorithm, a greedy approach that segments text by repeatedly choosing the longest possible valid term from a dictionary that matches the current position. The dictionary was expanded to include medical jargon, drug names, and local abbreviations to optimize tokenization accuracy for clinical narratives.

Formally, given an input string S and a dictionary D, at each position j, the algorithm identifies all candidate substrings of S beginning at j that appear in D, and selects the candidate with the maximum length. This substring is then appended to the output sequence of tokens, and the process continues from the next unmatched character until the entire string is segmented.

This left-to-right longest-match strategy enables more accurate tokenization of Thai medical terms.

The custom dictionary D integrated five sources (Figure 1) (1) English WordNet; (2) Thai WordNet; (3) SNOMED CT descriptions (providing English clinical terms and medical jargon); (4) Thai medicine terminology (the national medicine database contains drug ingredients, generic names, trade names, and herbal medicine); and (5) a manually curated abbreviation dictionary (Multimedia Appendix 1), comprising abbreviations with ≥2 capital letters occurring >100 times in the dataset. These abbreviations were reviewed by physicians, validated using 5 random samples of medical records, and mapped to SNOMED CT. This custom dictionary was also used to spell-check before tokenization, ensuring that incorrect or nonstandard spellings were corrected before segmentation (Figure 1).

The N-grams of length 1‐5, representing continuous sequences of N tokens (or grams), were generated to identify meaningful patterns and construct candidate terms or phrases.

For example, words contained unigram to 5-grams were: “ไข้” (fever) for unigram, “ปวด ท้อง” (abdominal pain) for bigram, “แผล ใน ปาก” (oral cavity wound) for trigram, “ขา สอง ข้าง บวม” (both legs are swelled) for 4-gram, and “เลือด ออก ผิดปกติ ทาง ช่องคลอด” (abnormal bleeding from vagina) for 5-gram. Syntactically irrelevant N-grams (ie, those containing >50% stop words, punctuation, or numbers; or terms beginning or ending with stop words or prepositions) were excluded. The remaining N-grams were ranked by frequency and carefully reviewed by experienced clinicians. Clinically relevant terms were defined as those that conveyed sufficient meaning for documenting patient conditions.

**Figure 2.**
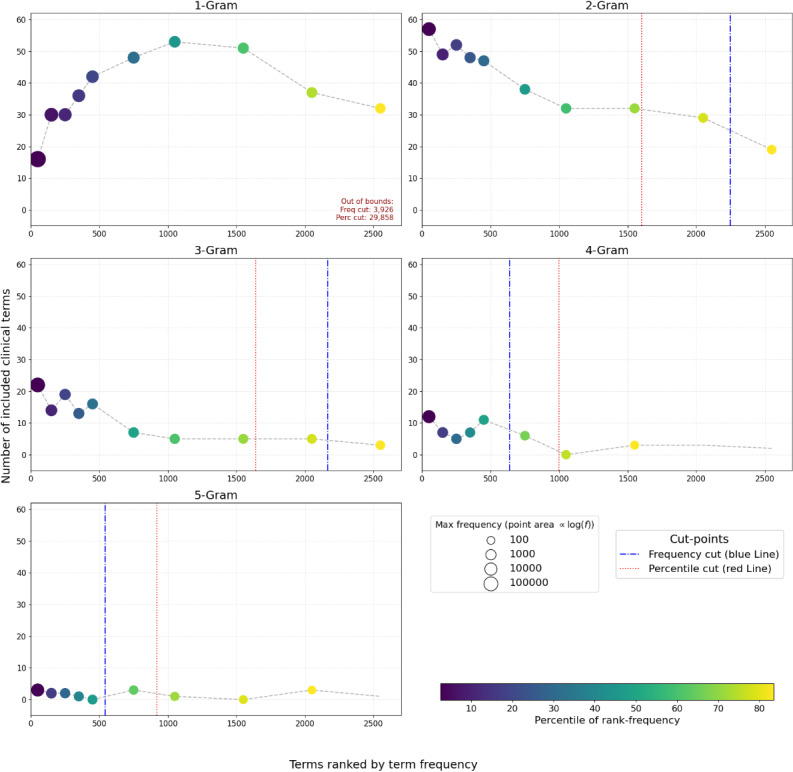
Numbers of included clinical terms per interval of rank-frequency categorized by the N of N-gram. Blue vertical lines indicate the cut point where term frequency was 300. Red vertical lines indicate the cut point where the percentile of rank-frequency reaches 70.

In the first round, medical doctors reviewed intervals of ranked lists (Multimedia Appendix 1), marked the terms that are clinically meaningful and appropriate for representing as structured data, and selected the terms by applying the following inclusion criteria: (1) frequency rank within the top 70th percentile or (2) absolute frequency ≥300 occurrences, thresholds that reflected diminishing returns in identifying clinically relevant terms (Figure 2). After the second round, 2054 terms were selected from the pool of 10,260 candidate terms for inclusion in the interface terminology, representing commonly used clinical expressions across EMRs.

### Mapping to SNOMED CT

Each UIT term was mapped to a semantically equivalent SNOMED CT concept using Snap2SNOMED, an open-source, publicly available mapping tool developed and maintained by SNOMED International. The tool provides key functionalities, including a built-in SNOMED CT browser, versioning, and automated lexical mapping (applicable to English-language terms only). Because automated mapping requires English input, 946 Thai terms were translated into English using Google Translate (Google LLC), and these translated terms were used as source terms for mapping. The automated mapping results were manually reviewed and validated by 2 clinical domain experts (NT and JS). To ensure semantic equivalency to the original Thai terms, 5 clinical usage contexts were supplied to the mappers for each term, selected at random from different patients. Manual mappings were guided by semantic equivalence criteria recommended in the Snap2SNOMED User Guide, prioritizing alignment for meaning, clinical intent, and hierarchical appropriateness. Disagreements between mappers were resolved through iterative discussion and consensus-based remapping.

Mapping results were classified into three categories, as illustrated in Figure 1: (1) fully mapped: SNOMED CT concepts that fully captured the semantics of the source term (eg, “valvular heart disease” mapped to “Heart valve disorder [disorder]”); (2) partially mapped: SNOMED CT concepts that were close but not fully semantically equivalent to the source term (eg, “home SBP” mapped to the narrower concept “Average home systolic blood pressure [observable entity]”); and (3) unmapped: source terms that lacked sufficient representation in existing SNOMED CT concepts.

A single source term could be potentially mapped to multiple SNOMED CT concepts (referred to as one-to-many mapping), resulting in more mapping pairs than source terms. This arose from one of two scenarios (1) homonyms, terms carried multiple distinct meanings, each represented by different SNOMED CT concepts following guidelines from SNOMED CT managing conventions and (2) ambiguous terms explicitly excluded from SNOMED CT due to insufficient contextual specificity (eg, “fundus” referring to either a hollow organ apex or the eye’s interior surface). These ambiguous terms were conservatively classified as unmapped to preserve terminological clarity and precision.

Partially mapped pairs were further classified into broader, narrower, and inexact relationships to quantify mapping quality. The broader referred to the SNOMED CT concept, which encompasses the meaning of the source term. Narrower referred to when the SNOMED CT concept represented only part of the source term’s meaning. In cases where semantic equivalence required postcoordination with multiple SNOMED CT concepts, we selected a single precoordinated concept in the same hierarchy as the narrower one. For example, “nodule at right upper lung” was mapped to the broader concept *“*Nodule of lung (disorder)*”* rather than combining a finding with “Upper lobe of right lung (body structure).” Inexact referred to mappings where the best available SNOMED CT concept differed in lexical and semantic characteristics but provided a clinically relevant approximation. For example, “over-the-counter drug use” was inexactly mapped to “Patient on self-medication (finding).”

### Mapping Analysis

Terms unable to be mapped by mappers were discussed and categorized into either partially mapped or unmapped terms, including ambiguous terms and terms for which no semantic equivalency was found in SNOMED CT. For the terms that can be represented using postcoordination of 2 or more concepts from the same hierarchy, for example, “เหงื่อแตกใจสั่น” (sweating and palpitation), we decided to categorize them as unmapped terms for further analysis. Partially mapped and unmapped terms were analyzed to assess their potential for practical implementation within our research team. Specifically, the analysis examined whether these terms could be adequately represented through SNOMED CT postcoordination or should be proposed for inclusion in an updated national edition of SNOMED CT. This evaluation helped identify specific content areas where concept expansion would better align the terminology with local clinical practice and ensure comprehensive coverage in the national edition.

### Ethical Considerations

The study protocol was reviewed and approved by the Ramathibodi Human Research Ethics Committee (Approval number COA.MURA2023/469). The informed consent was waived due to the retrospective nature of the dataset and minimal risk to participants. All personally identifiable information (eg, names, surnames, dates of birth, addresses, hospital numbers, and visit dates) was removed from the dataset prior to analysis to ensure privacy and confidentiality.

## Results

### Dataset Characteristics

A total of 1,88,468 documents were initially retrieved from the EMRs during the study period. After excluding records with missing data, 1,83,557 documents from 53,142 patients were included in the analysis to develop the interface terminology. Characteristics of the 53,142 patients are described in Table 1. IM patients were the oldest (mean 59.3, SD 16.9), followed by FM (mean 54.4, SD 15.2), and emergency department (mean 43.4, SD 28.0). The percentage of males was highest in emergency (44.3%), followed by IM (39.7%) and FM (36.94%).

At the document level, the IM department contributed the largest volume (1,18,382/1,83,557, 64.5%), followed by the emergency (34,829/1,83,557, 19.0%) and FM departments (30,346/1,83,557, 16.5%). The median number of documents per patient (IQR) was 2 (1-5) for IM, 1 (1-2) for emergency, and 1 (1-3) for FM (Table 1).

Text-level analysis of 1,83,557 documents yielded 14,946,559 total words. IM contributed the majority (10,471,768/14,946,559, 70.1%), followed by emergency (2,750,896/14,946,559, 18.4%) and FM (1,723,895/14,946,559, 11.5%). Median (IQR) words per document were 107 (62-175) for IM, 74 (44-125) for ER, and 60 (38-99) for FM. Thai words were varied by department. Emergency department documents had the highest proportion of Thai words (1,752,756/2,750,896, 63.7%), followed by FM (1,016,914/1,723,895, 59.0%), while IM contained the lowest proportion (4,154,255/10,471,768, 39.7%).

**Table 1.** Characteristics of data from the 3 outpatient departments.

Characteristics	FM^a^	ER^b^	IM^c^	Total
Patient-level				
Number of patients (n)	12,128	22,878	29,985	53,142
Age (years), mean (SD)	54.37 (15.21)	43.42 (27.97)	59.31 (16.91)	53.98 (15.42)
Male (sex), n (%)	4480 (36.94)	10,137 (44.31)	11,892 (39.66)	21,821 (41.06)
Document level				
Number of documents (n)	30,346	34,829	1,18,382	1,83,557
Documents per patient, median (IQR)	1 (1-3)	1 (1-2)	2 (1-5)	2 (1-4)
Text-level				
Unique words (n)	20,070	25,055	33,286	41,755
Unique Thai words, n (%)	10,475 (52.19)	12,647 (50.78)	15,216 (45.71)	20,407 (48.87)
Total words (n)	1,723,895	2,750,896	10,471,768	14,946,559
Total Thai words, n (%)	1,016,914 (58.99)	1,752,756 (63.72)	4,154,255 (39.67)	6,923,925 (46.32)
Word per document, median (IQR)	60 (38‐99)	74 (44‐125)	107 (62‐175)	81 (43‐141)

a FM: family medicine.

b ER: emergency room.

c IM: internal medicine.

### Coverage

A total of 2054 frequently used clinical source terms met the inclusion criteria and were integrated into the interface terminology. Of these, 2012 terms were successfully mapped to 2041 SNOMED CT concepts. Most terms were mapped one-to-one (1984 terms), while 28 terms were mapped to 57 multiconcepts through one-to-many mappings, yielding a total of 2083 entries while covering 1486 unique SNOMED CT codes. The final mappings are provided in Multimedia Appendix 2. Mapping characteristics are as follows:

- Language: English (1140 out of 2083 terms [54.73%]) and Thai (943 out of 2083 terms [45.27%]).
- N-gram: unigrams (1098 terms), bigrams (741 terms), trigrams (189 terms), 4-grams (45 terms), and 5-grams (10 terms).
- Equivalency: 1781 (85.50%) fully mapped terms, 123 (5.90%) mapped to broader concepts, 56 (2.69%) mapped to narrower concepts, and 81 (3.89%) inexactly mapped terms (Table 2). The remaining 42 terms (2.02%) could not be mapped due to culturally specific expressions or clinical concepts not yet represented in any SNOMED CT concept.
- Overall, the concept coverage was 85.50%. Combining fully and partially mapped terms yielded an overall concept coverage of 97.98%.

Term coverage is defined as the percentage of local clinical terms that can be accurately mapped to an existing SNOMED CT description. Among 1140 English terms, 656 terms (31.49%) had direct lexical matches with English SNOMED CT descriptions, including simple variations of writing, for example, “wound dressing” versus “Dressing wound.” The remaining 484 English terms (23.24%) did not have an exact lexical match and were still mappable through contextual interpretation, such as local abbreviations (eg, “f/u” *→* “Follow-up visit [procedure]”), or variation in writing style (eg, “contact tb” → “Exposure to tuberculosis [situation]”). As expected, no Thai terms were considered direct lexical matches because SNOMED CT descriptions are not yet translated into Thai.

**Table 2.** Concept coverage of Systematized Nomenclature of Medicine Clinical Terms (SNOMED CT) for Thai interface terminology.

Concept equivalency	English	Thai	Total
Fully mapped, n (%)	973 (46.71)	808 (38.79)	1781 (85.50)
Partially mapped, n (%)	140 (6.72)	120 (5.75)	260 (14.48)
Broader	65 (3.12)	58 (2.78)	123 (5.90)
Narrower	38 (1.82)	18(0.86)	56 (2.69)
Inexact	37 (1.78)	44 (2.11)	81 (3.89)
Unmapped, n (%)	27 (1.30)	15 (0.72)	42 (2.02)
Total, n (%)	1140 (54.73)	943 (45.27)	2083 (100)

### Mapping Analysis

Mapping analysis of the 260 partially mapped terms revealed linguistic and semantic challenges. All 123 broader (5.90%) and 56 narrower (2.69%) pairs comprised of concepts differed in granularity, where terms belonged to the same semantic domain, but they were at different levels of clinical detail or abstraction. On the other hand, inexact terms arose when clinical intention aligned, but lexical or cultural expression differed. For example, “นอนหนุนหมอน” (sleep with multiple pillows) was used to indicate the situation when a patient cannot sleep lying down due to shortness of breath; it was inexactly mapped to *“*Orthopnea (finding)” and “ทานยาสม่ำเสมอ (taking medicine regularly)” and its variations were the patient-reported concept “Drug compliance good (finding).”

We also observed that culture and linguistic characteristics contributed to mismatches. Some Thai expressions convey complex clinical scenarios through idiomatic phrasing, complicating alignment with SNOMED CT. For example, “over-the-counter drug use” in the Thai context often implies access to medications that would require prescriptions in many other countries. After deliberation in our team, although this was mapped to “Patient on self-medication (finding),” this concept does not fully capture its implications within Thailand’s health care system.

Forty-two unmapped terms (2.02%) were further categorized into 3 categories based on their characteristics (see Table 3).

Ambiguous terms (23/42, 54.76%) violated SNOMED CT naming conventions and required additional contexts for appropriate representation (eg, “lobe,” “node,” and “septum”).

Multiple clinical concepts (10/42, 23.81%) represented multiple distinct findings or events (eg, “s1 s2” [heart sounds], “เหงื่อแตกใจสั่น” [sweating and palpitation], “ล้มศีรษะกระแทก” [fall and head collision]). Many terms reflect Thai-specific linguistic constructions and could potentially be addressed through postcoordination.

Clinically meaningful terms without SNOMED CT equivalents (9/42, 21.43%). Some terms were (locally) nationally specific (eg, “หวิว” [fear of heights or a feeling of butterflies in the stomach]) while others were universally applicable but absent from SNOMED CT (eg, “ยาลดไข้” [antipyretic medicine] and “DOI” [date of infection]). The terms were exhaustively searched using both lexical and graph properties of SNOMED CT through the use of SNOMED CT Expression Constraint Language.

**Table 3.** Categories for unmapped concepts.

Characteristics	Ambiguous terms	Multiconcept terms	No concept terms
Value, n (%)	23 (54.76)	10 (23.81)	9 (21.43)
Further intervention	Exclusion from terminology or addition of contextual information	Postcoordination or separation to multiple distinct concepts	Creation of new concept
Examples	Lobe Node Septum	S1S2 (Heart sounds) เหงื่อแตกใจสั่น (sweating and palpitation) ล้มศีรษะกระแทก (fall and head collision)	DOI (Date of Infection) ยาลดไข้ (Antipyretic medicine) หวิว (fear of heights or a feeling of butterflies in the stomach)

## Discussion

### Principal Findings

This study developed a multilingual UIT derived from real-world clinical documents across the FM, emergency, and IM departments and evaluated its alignment with SNOMED CT. The resulting UIT reflects a nearly balanced linguistic composition (54.49% English terms and 45.51% Thai terms). Consistent with the bilingual nature of Thai medical records. SNOMED CT demonstrated strong concept coverage with 85.50% full and 97.98% full plus partial coverage, confirming its suitability as a reference terminology even in the setting where nearly half of the source terms are non-English. However, term coverage remained modest (31.49%) because SNOMED CT descriptions have not yet been translated into Thai, making exact lexical matches possible only for English-language terms.

The source data also showed different patterns in clinical documentation practices. For instance, IM generated the highest volume of text but had the lowest proportion of Thai words, consistent with its more technical and specialty-oriented clinical activities. In contrast, emergency and FM documents contained more Thai narrative language, which may reflect more direct patient-provider interaction, brief encounters, and patient-reported symptoms. These differences emphasize the need for UITs that can accommodate both biomedical language and colloquial Thai expressions.

We also found that only a small proportion of terms remained unmapped (2.02%), underscoring that the majority of local expressions can be integrated into SNOMED CT without major structural changes. These relatively small, unmapped concepts may help identify where efforts should be strategically concentrated at the national level. Moreover, the moderate-term coverage (31.49%), driven entirely by English lexical matches, reinforces the need for localized terminology tools and translation strategies if Thailand aims to scale SNOMED CT adoption nationwide.

### Comparison With Previous Work

Despite differences in term extraction methods, particularly our focus on commonly used clinical terms and the use of a more recent SNOMED CT release, our findings generally align with previous evaluations of SNOMED CT concept coverage. Prior studies have reported a strong preference for high coverage in the English-language international edition (86% fully mapped and 92% partially mapped) and similar strong performance in the fully translated Swedish edition when applied to Swedish clinical datasets (87% fully mapped and 92% partially mapped) [19]. In comparison, our findings showed 85.50% full and 97.98% full plus partial coverage, demonstrating that SNOMED CT remains robust even in bilingual and partially non-English documentation environments.

Comparable evidence has also been observed in low- and middle-income country settings. For example, an evaluation from Argentina reported 83% coverage based on user feedback during real-world implementation [12]. Other specific domains targeting nationwide data collection and surveillance objectives have very high coverage, 99% precoordinated and 100% postcoordinated concept coverage for AIDS-defining illnesses in Kenya [25], and high correspondence for tuberculosis-related terminology, although the latter did not include a formal concept coverage analysis [14].

In Thailand, a prior study mapped Thai discharge summary notes to SNOMED CT but removed non-English content and did not report concept coverage [21]. Our study extends this work by incorporating both English and Thai clinical terms from 3 departments, performing concept coverage, term coverage, and mapping analysis, and examining implications for national UIT development. This provides a more comprehensive and representative assessment of SNOMED CT’s applicability for Thai primary care and hospital settings.

Partially mapped terms frequently reflect homonyms (eg, “มวน” meaning both abdominal colic and a cigarette unit), mismatches in granularity (eg, “hypoglycemic symptom” mapped to hypoglycemia [disorder]), or culturally shaped usage patterns. These challenges are consistent with those reported in other multilingual SNOMED CT mapping studies [10,26]. In the Thai language, many biomedical concepts and terms are not readily expressed in Thai. Unlike languages that share linguistic roots (eg, English, French, and Swedish), Thai lacks direct lexical equivalents for some medical terms, creating communication barriers between clinicians, patients, and even health care providers. To ensure mutual understanding, clinicians need to phrase simple questions that patients can comprehend, allowing information to be collected accurately. For example, “compliance” is simply conveyed as “taking medicine regularly” or “never miss a dose,” which is reflected in how these terms appeared in the EMRs. This observation has also been reported in other languages, not from the same roots as the English language [27].

Term coverage in our dataset was modest (31.49%), driven entirely by English lexical matches. The observed term coverage is comparable with countries whose SNOMED CT editions are only partially translated, for example, Dutch (35%) and French (39%) [19]. A considerable proportion of our Thai clinical terms were synonymous with globally common English medical jargon, reflecting Thailand’s long-standing integration of English terminology in medical education and clinical communication. As shown in Table 1, Thai language use was more prevalent in primary care departments (FM and emergency), while IM documents contained more specialized English terminology, consistent with previous reports [28].

### Challenges in Expressing Clinical Concepts in the Thai Context

Our mapping analysis of partially mapped terms highlighted several challenges in representing Thai clinical concepts within SNOMED CT. Many partially mapped terms require determining whether they should (1) be represented through postcoordination with existing SNOMED CT concepts (thereby supporting international interoperability) or (2) be introduced as new concepts in the Thai national extension to preserve locally specific meaning.

Postcoordination can be beneficial for international interoperability but may lose nuanced local semantics. Conversely, creating new concepts preserves the locally used expression but introduces a long-term maintenance burden and requires clear criteria for inclusion. No standardized national guidelines currently exist; decisions must consider clinical relevance, documentation frequency, clinical importance, and anticipated use cases within Thailand’s EMR ecosystem. A representative example from our study is “over-the-counter drug use,” which frequently appeared in our dataset and reflects a long-standing public health challenge in unsupervised medication access in Thailand [29], which contributes to national issues such as antimicrobial resistance and drug misuse [30]. While “Patient on self-medication (finding)” is the most relevant concept in SNOMED CT, it does not fully represent Thailand’s regulatory distinctions and safety concerns. If more granular differentiation is required in Thailand for surveillance or policy purposes, a new child concept under *“*Patient on self-medication (finding)*”* may be warranted.

The analysis of unmapped terms also highlighted the role of language-specific and culturally specific expressions that are not directly translatable into English (see Table 3). In our data, 20.93% of unmapped terms reflected culturally unique, ambiguous, or context-dependent meanings. These findings suggested three key strategies for terminology management and strengthening (1) exclude ambiguous terms or add contextual information to the context-dependent terms. For example, the Thai term “หวิว” has multiple meanings (fear of heights or a feeling of butterflies in the stomach). Instead of mapping it directly, adding the more specific term “ใจหวิว” (giddiness in the heart) mapped to “Palpitations (finding)” can ensure clarity and avoid misclassification. (2) Use postcoordination for multiconcept or idiomatic expressions. This pattern was also found in the English context, for example, the concept “Nausea and vomiting (finding),” which exists as a precoordinated concept within the international edition of SNOMED CT. However, since separated distinct concepts are presented, we suggest mapping to separate entities to preserve legacy information and also interoperability with the international context. (3) Recommend new concepts for national extension or international inclusion for globally relevant but unsupported terms. This should be done with a well-established concept with clear practical implications. One example we found was a local abbreviation, “DOI (Date of Infection),” which we believe has seen an increase in occurrences during the pandemic, particularly in cases where infectious history has been thoroughly collected. Additionally, some of these represent global clinically relevant concepts insufficiently addressed by SNOMED CT, indicating areas for future expansion [7].

Overall, these challenges highlight the need for national governance structures, clearly defined criteria for new concept creation, and a systematic strategy for translation and localization to ensure accurate, consistent, and interoperable representation of Thai clinical terminology. Regional guidance from the Asia eHealth Information Network (AeHIN) similarly emphasizes that successful and sustainable interoperability depends not only on the technical standards but also on governance, architecture, workforce capacity, and institutional processes [31]. Centralized governance through a national health data dictionary and terminology services, as described in many interoperability architectures, like Open Health Information Exchange (OpenHIE; Linux Foundation Public Health), can standardize terminological assets and reduce fragmentation across implementation sites [32]. Such services can support the controlled use of postcoordination and governed concept creation and enable local expressiveness while preserving semantic consistency and international interoperability. Importantly, these functions are best managed at the terminology service level rather than within individual EMR implementations, thereby reducing duplication and implementation burden. In addition, mappings between shared UITs and reference terminologies or classification systems (eg, the *ICD* family) should be centrally maintained, versioned, and routinely updated. For example, the OpenMRS ecosystem also adopts the similar implementation pattern, where many deployments rely on the CIEL interface terminology, centrally managed and distributed via Open Concept Lab, supporting reuse and reducing divergence across implementations [13].

### Strengths and Limitations

To our knowledge, this is the first study in Thailand—and among the few resource-limited countries—to develop a UIT and evaluate both concept- and term-level coverage of SNOMED CT in a real-world clinical setting. A key strength of this study lies in its data source, free-text entries from actual clinical encounters, which ensures that the extracted terms are grounded in real-world usage. Moreover, the terminology development process integrated clinical expertise with NLP techniques in a resource-limited setting, offering valuable insights for similar environments. The final UIT is composed of the commonly used clinical term. Although it is small in numbers, we believe this terminology to be generally applicable for use within Thailand. Although SNOMED CT concept coverage has been examined in a few resource-limited country settings, evidence remains scarce. This study extends the discussion to Southeast Asia by evaluating Thai-language primary care data, constructing a UIT mapped to SNOMED CT, and reporting concept coverage.

Several limitations should be acknowledged. First, source data were collected from a single hospital, which may limit the generalizability of the developed UIT. Consequently, the developed UIT may not fully capture the diversity of expression terms used across Thailand’s broader health care system, particularly in regions where distinct dialects or documentation styles prevail. Nevertheless, this study achieved its goal of constructing a realistic UIT to inform national implementation strategies and evaluate the feasibility of adopting SNOMED CT as a national standard. As new clinical terms and concepts emerge, the UTI can be continuously expanded through routine monitoring, domain-expert review, and integration of automated extraction tools to ensure incremental improvement over time. Second, although the mapping and term extraction processes were performed by clinically trained physicians, the subjective nature of manual mapping introduces the potential for interpretation bias or human error. An interannotator agreement score was not assessed due to constraints in available personnel and resources; consequently, only one mapper and one reviewer contributed to the final mapping. Third, the coverage results may be overestimated compared to those reported in previous studies because our term list was developed from commonly used clinical expressions and mapped to a more recent SNOMED CT release. At the same time, the mapping tool Snap2SNOMED does not support postcoordination at the time of this writing, which may lead to a potential underestimation of full SNOMED CT content coverage. However, since postcoordination has not been generally performed in previous studies, the results remain broadly comparable with existing evidence [19].

### Recommendation for Future Research

Future research should expand the evaluation of concept and term coverage across hospitals of varying sizes and settings, including northern, northeastern, and southern regions of Thailand, where specific dialects may influence clinical documentation practices. Extending these methods beyond a single institution will help determine the generalizability of the UIT and support efforts toward national standardization.

Automated methods should also be explored. Advanced NLP techniques and large language models may substantially improve the efficiency and accuracy of term extraction, disambiguation, and SNOMED CT mapping, particularly as our team is already beginning to implement these tools in parallel methodological work. Additional studies should examine how postcoordination can be operationalized in real-world EMRs, with the goal of identifying best practices that maintain semantic precision without burdening clinicians during documentation.

Finally, future research should include more diverse document types (eg, operative notes, procedure reports, and specialty consults) and a broader population. Doing such improves characterization of locally used clinical concepts, strengthens coverage estimates, and supports the development of a more comprehensive national UIT that accurately reflects the linguistic and clinical diversity of Thailand’s health care system.

### Conclusions

In conclusion, SNOMED CT represents most common clinical concepts but lacks sufficient local language coverage. With the interpretation of local clinical terms, a Thai UIT mapped to SNOMED CT can serve as a comprehensive standard for clinical documentation. This hybrid approach can enhance data quality, promote interoperability, and facilitate better use of health care data, contributing to improved patient care and creating opportunities for international collaboration.

## Supplementary material

10.2196/80039 Multimedia Appendix 1 Top 10 frequency of the most common abbreviations and First-round results of interface term selection.

10.2196/80039 Multimedia Appendix 2 Interface Terminology mapped to SNOMED CT (Systematized Nomenclature of Medicine Clinical Terms).

## Abbreviations

AeHIN: Asia eHealth Information Network
CIEL: Columbia International eHealth Laboratory
EMR: electronic medical record
FM: family medicine
*ICD*: *International Classification of Diseases*
IM: internal medicine
NLP: natural language processing
OpenHIE: Open Health Information Exchange
OpenMRS: Open Medical Record System
SNOMED CT: Systematized Nomenclature of Medicine Clinical Terms
UIT: user interface terminology
