# Supplementary material for: Developing a Thai User Interface Terminology for Systematized Nomenclature of Medicine Clinical Terms Implementation in Primary Care: Cross-Sectional Content Coverage Analysis
Source: JMIR Med Inform. 2026 Mar 9;14:e80039. doi: 10.2196/80039 (PMC12978892; doi:10.2196/80039)
Supplement: Multimedia Appendix 1 [file medinform-v14-e80039-s001.docx]

Supplementary tables

table 1 Top 10 frequency of the most common abbreviations

table 2 First round results of interface term selection

1. unigram
2. bigram
3. trigram
4. four-gram
5. five-gram

Supplement table 1 Top 10 frequency of the most common abbreviations

| words | Frequency | longform | SCTID | FSN | term coverage | concept coverage |
| --- | --- | --- | --- | --- | --- | --- |
|  |  |  |  |  |  |  |
| dlp | 64546 | Dyslipidemia | 370992007 | Dyslipidemia (disorder) | 0 | TARGET_EQUIVALENT |
| cxr | 31668 | Chest X Ray | 399208008 | Plain chest X-ray (procedure) | 1 | TARGET_EQUIVALENT |
| cbc | 28556 | Complete blood count | 26604007 | Complete blood count (procedure) | 1 | TARGET_EQUIVALENT |
| ua | 26412 | Urine Analysis | 27171005 | Urinalysis (procedure) | 0 | TARGET_EQUIVALENT |
| wk | 25822 | Week | 258705008 | week (qualifier value) | 1 | TARGET_EQUIVALENT |
| hx | 25014 | History | 392521001 | History of (contextual qualifier) (qualifier value) | 0 | TARGET_EQUIVALENT |
| hct | 24430 | Hematocrit | 28317006 | Hematocrit determination (procedure) | 1 | TARGET_EQUIVALENT |
| wnl | 20255 | Within Normal Limits | 260394003 | Normal limits (qualifier value) | 0 | TARGET_EQUIVALENT |
| plt | 16493 | Platelet | 16378004 | Platelet (cell structure) | 1 | TARGET_EQUIVALENT |
| rm | 15769 | Repeat medication | 414938004 | On repeat dispensing system (finding) | 0 | TARGET_INEXACT |

Supplement table 2 First round results of interface term selection

| Index | Frequency  (max-min) | Percentile  (max-min) | Positive concept |
| --- | --- | --- | --- |
| a) unigram | | | |
| 1-100 | 1,819,656 – 32,901 | 1 – 4 | 6 |
| 101-200 | 32,709 – 15,372 | 4 - 8 | 30 |
| 201-300 | 15,203 – 9,831 | 8 - 12 | 30 |
| 301-400 | 9,797 – 7,352 | 12 - 16 | 36 |
| 401-500 | 7,324 – 5,741 | 16 – 20 | 42 |
| 701-800 | 3,853 – 3,227 | 27 - 31 | 48 |
| 1001-1100 | 2,346 – 2,048 | 39 - 42 | 53 |
| 1501-1600 | 1,365 – 1,236 | 53 - 56 | 51 |
| 2001-2100 | 922 - 860 | 66 - 67 | 37 |
| 2501-2600 | 657 – 609 | 74 - 76 | 32 |
| b) bigram | | | |
| 1-100 | 33,241-3,497 | 1-8 | 57 |
| 101-200 | 3,459-2,128 | 8-15 | 49 |
| 201-300 | 2,101-1,549 | 15-22 | 52 |
| 301-400 | 1,549-1,249 | 22-28 | 48 |
| 401-500 | 1,244-1,067 | 28-33 | 47 |
| 701-800 | 802-726 | 44-48 | 38 |
| 1001-1100 | 612-567 | 56-59 | 32 |
| 1501-1600 | 435-413 | 68-70 | 32 |
| 2001-2100 | 339-324 | 75-76 | 29 |
| 2501-2600 | 272-263 | 80-81 | 19 |
| c) trigram | | | |
| 1-100 | 147,287-2,594 | 1-9 | 22 |
| 101-200 | 2,560-1,626 | 9-16 | 14 |
| 201-300 | 1,624-1,232 | 17-23 | 19 |
| 301-400 | 1,228-1,034 | 23-30 | 13 |
| 401-500 | 1,029-886 | 30-36 | 16 |
| 701-800 | 699-633 | 46-50 | 7 |
| 1001-1100 | 543-500 | 57-60 | 5 |
| 1501-1600 | 405-387 | 67-69 | 5 |
| 2001-2100 | 322-309 | 74-75 | 5 |
| 2501-2600 | 265-256 | 79-80 | 3 |
| d) four-gram | | | |
| 1-100 | 13,232-937 | 1-15 | 12 |
| 101-200 | 937-627 | 15-27 | 7 |
| 201-300 | 627-489 | 27-37 | 5 |
| 301-400 | 489-412 | 37-45 | 7 |
| 401-500 | 409-359 | 45-51 | 11 |
| 701-800 | 276-248 | 61-65 | 6 |
| 1001-1100 | 217-203 | 70-72 | 0 |
| 1501-1600 | 164-156 | 77-78 | 3 |
| 2001-2100 | 133-128 | 82 | 3 |
| 2501-2600 | 113-110 | 85 | 2 |
| e) five-gram | | | |
| 1-100 | 1-100 | 1-100 | 1-100 |
| 101-200 | 101-200 | 101-200 | 101-200 |
| 201-300 | 201-300 | 201-300 | 201-300 |
| 301-400 | 301-400 | 301-400 | 301-400 |
| 401-500 | 401-500 | 401-500 | 401-500 |
| 701-800 | 701-800 | 701-800 | 701-800 |
| 1001-1100 | 1001-1100 | 1001-1100 | 1001-1100 |
| 1501-1600 | 1501-1600 | 1501-1600 | 1501-1600 |
| 2001-2100 | 2001-2100 | 2001-2100 | 2001-2100 |
| 2501-2600 | 2501-2600 | 2501-2600 | 2501-2600 |
